# Supplementary material for: Identifying the Pathogenic Variants in Heart Genes in Vietnamese Sudden Unexplained Death Victims by Next-Generation Sequencing
Source: Diagnostics (Basel). 2024 Aug 27;14(17):1876. doi: 10.3390/diagnostics14171876 (PMC11394071; doi:10.3390/diagnostics14171876)
Supplement: Supplementary file 1 [file diagnostics-14-01876-s001.zip › diagnostics-3137086-supplementary.pdf]

Table S1. List of 167 genes in panel gene was used in this study

Including genes associated with arrhythmia and cardiomyopathy:

AARS2, ABCC6, ABCC9, ACADVL, ACTA1, ACTC1, ACTN2, AGK, AGL, AKAP9, ALMS1, ANK2, ANO5, APOA1, BAG3, BRAF, CACNA1C, CACNA1D, CACNA2D1, CACNB2, CALM1, CALM2, CALM3, CALR3, CAPN3, CASQ2, CAV3, CBL, COX15, CPT2, CRYAB, CSRP3, CTF1, DBH, DES, DMD, DNAJC19, DOLK, DSC2, DSG2, DSP, DTNA, DYSF, EMD, ETFA, ETFB, ETFDH, EYA4, FBN2, FHL1, FKPP, FKTN, FLNC, FOXRED1, FXN, GAA, GATAD1, GATA6, GBE1, GDP1L, GFM1, GLA, GLB1, GSN, GUSB, HADHA, HCN4, HFE, HRAS, HTRA1, ISPD, JPH2, JUP, KCNA5, KCNE1, KCNE2, KCNE3, KCNE4, KCNE5, KCND3, KCNH2, KCNJ2, KCNJ5, KCNJ8, KCNQ1, KRAS, LAMA2, LAMA4, LAMP2, LDB3, LMNA, MAP2K1, MAP2K2, MLYCD, MTO1, MYBPC3, MYH6, MYH7, MYL2, MYL3, MYLK, MYOT, MYOZ2, MYPN, NEBL, NEXN, NF1, NKX2-5, NRAS, PCCA, PCCB, PKP2, PLEC, PNPLA2, PRDM16, PRKAG2, PSEN1, PSEN2, PTPN11, RAF1, RBM20, RMND1, RYR2, SALL4, SCN1B, SCN2B, SCN3B, SCN4B, SCN5A, SCN10A, SCNN1B, SCNN1G, SCO2, SDHA, SGCA, SGCB, SGCD, SGCG, SHOC2, SLC4A3, SLC22A5, SLC25A4, SLC25A20, SOS1, SPRED1, TAB2, TAZ, TBX5, TBX20, TCAP, TGFB3, TMEM43, TMEM70, TMPO, TNNC1, TNNI3, TNNI3K, TNNT2, TPM1, TRDN, TRIM32, TRPM4, TSFM, TTN, TTR, VCL, VPS13A.

Table S2. Prediction results using *in silico* tools for splicing variant *SLC4A3:* c.2535+1G>A

MaxEntScan

>widtype

AAGGTGGAG	MAXENT: 1.51	MDD: 8.38	MM: 3.88	WMM: 4.57

>mutant

AAGATGGAG	MAXENT: -6.67	MDD: 0.20	MM: -4.30	WMM: -3.62

NetGene2 v. 2.4

The sequence: **wildtype** has the following composition:
Donor splice sites, direct strand

pos 5'->3'	phase	strand	confidence	5' exon intron 3'	
159	0	+	1.00	CTTCTTCAAG^GTGAGGCGAA	H
**583**	0	+	0.60	GCTCTACAAG^GTGGAGGTCC	
599	1	+	0.19	GTCCAGCGAG^GTCTTGGGGG	
662	0	+	0.34	GGAGGGGGAG^GTATGGAGAA	
768	1	+	0.34	GACCCAGGGG^GTGGGTGCCA	
834	2	+	0.46	ACTTGCACAG^GTATGTGATG	

The sequence: **mutant** has the following composition:
Donor splice sites, direct strand

pos 5'->3'	phase	strand	confidence	5' exon intron 3'	
159	0	+	1.00	CTTCTTCAAG^GTGAGGCGAA	H
599	1	+	0.19	GTCCAGCGAG^GTCTTGGGGG	
662	0	+	0.34	GGAGGGGGAG^GTATGGAGAA	
768	1	+	0.34	GACCCAGGGG^GTGGGTGCCA	
834	2	+	0.46	ACTTGCACAG^GTATGTGATG	

Spliceailookup

SpliceAI scores: ?

Variant	Gene (□ = MANE Select transcript □ = non-coding transcript)	Δ type	Δ score	position	REF score	ALT score
chr2-219636875-G-A splice donor variant UCSC, gnomAD	SLC4A3 (ENSG00000114923.17 / ENST00000358055.8 / NM_005070.4) protein coding MANE Select transcript (plus strand) OMIM, GTEx, gnomAD, ClinGen, Ensembl, Decipher, GeneCards	Acceptor Loss	0.00	-255 bp	0.14	0.12
		Donor Loss	0.98	-1 bp	0.98	0.00
		Acceptor Gain	0.07	-63 bp	0.22	0.30
		Donor Gain	0.73	5 bp	0.12	0.85

MANE Select Transcript or All Transcripts

Pangolin scores: ?

Variant	Gene	Δ type	Δ score	position	REF score	ALT score
chr2-219636875-G-A splice donor variant UCSC, gnomAD	SLC4A3 (ENSG00000114923.17 / ENST00000358055.8 / NM_005070.4) protein coding MANE Select transcript (plus strand) OMIM, GTEx, gnomAD, ClinGen, Ensembl, Decipher, GeneCards	Splice Loss	0.80	-1 bp	0.87	0.07
		Splice Gain	0.37	5 bp	0.13	0.49

EX-SKIP - Results for submitted sequences

Seq	PESS (count)	FAS-ESS hex2 (count)	FAS-ESS hex3 (count)	IIE (count)	IIE (sum)	NI-ESS trusted (count)	NI-ESS all (sum)	PESE (count)	RESCUE -ESE (count)	EIE (count)	EIE (sum)	NI-ESE trusted (count)	NI-ESE all (sum)	ESS (total)	ESE (total)	ESS/ESE (ratio)
SLC4A3	9	102	58	354	6124.2256	157	-218.8672	50	34	312	4030.8882	258	370.1007	680	654	1.04

Allele SLC4A3 has a higher chance of exon skipping than allele .

Fruitfly

Donor site predictions for wildtype:

Start	End	Score	Exon Intron
152	166	**0.99**	cttcaaggtgaggcg
655	669	0.44	gggggaggtatggag
827	841	0.99	tgcacaggtatgtga
926	940	0.55	gtgtggtgtgagtat
951	965	0.53	gtgtgatgtatgtgt

Acceptor site predictions for wildtype:

Start	End	Score	Intron Exon
251	291	0.87	cccacactcttccttacctaggggatgggtccctgcattct
307	347	0.84	ttcatccgctgcctattccaggggcattgacacccagggc
367	407	0.74	accgctcctacccccacctagttctgccgagcccaggacct
499	539	0.42	tacatctcgcctttcacccaggagatctttgcctttctcat
877	917	0.64	tccttgggtcaccttttgtagaggagtgtgtgtgtgtgtgt

Donor site predictions for mutant:

Start	End	Score	Exon Intron
152	166	0.99	cttcaaggtgaggcg
655	669	0.44	gggggaggtatggag
827	841	0.99	tgcacaggtatgtga
926	940	0.55	gtgtggtgtgagtat
951	965	0.53	gtgtgatgtatgtgt

Acceptor site predictions for mutant:

Start	End	Score	Intron Exon
251	291	0.87	cccacactcttccttacctaggggatgggtccctgcattct
307	347	0.84	ttcatccgctgcctattccaggggcattgacacccagggc
367	407	0.74	accgctcctacccccacctagttctgccgagcccaggacct
499	539	0.42	tacatctcgcctttcacccaggagatctttgcctttctcat
877	917	0.64	tccttgggtcaccttttgtagaggagtgtgtgtgtgtgtgt
